# Association between dietary protein intake and changes in health-related quality of life in older adults: findings from the AusDiab 12-year prospective study

**DOI:** 10.1186/s12877-022-02894-y

**Published:** 2022-03-16

**Authors:** Annabel P. Matison, Catherine M. Milte, Jonathan E. Shaw, Dianna J. Magliano, Robin M. Daly, Susan J. Torres

**Affiliations:** 1 Deakin University, Institute for Physical Activity and Nutrition, 221 Burwood Highway, Burwood, Victoria 3125 Australia; 2 Centre for Healthy Brain Ageing, University of New South Wales, School of Psychiatry, Level 1, AGSM (G27) Gate 11, Botany Street, Sydney, New South Wales 2052 Australia; 3 Baker Heart and Diabetes Institute, Level 4, 99 Commercial Road, Melbourne, Victoria 3004 Australia

**Keywords:** Dietary protein, Quality of life, Longitudinal study, Older adults

## Abstract

**Background:**

Adequate dietary protein intake is recommended for older adults to optimise muscle health and function, and support recovery from illness, however, its effect on health-related quality of life (HRQoL) is unclear. The aim of this study was to examine the association between total protein intake and different sources of dietary protein and HRQoL in Australians aged 60 years and older over a 12-year period.

**Methods:**

This study used data from the Australian Diabetes, Obesity and Lifestyle study (AusDiab), a 12-year population-based prospective study. The sample included 752 (386 females) adults aged 60 years and older. Protein intake was estimated at baseline (1999/2000) from a 74-item Food Frequency Questionnaire, and HRQoL using the 36-item Short-form Health Survey assessed at baseline (1999/2000) and after 12 years (2011/12). The association between protein intake and change in HRQoL was evaluated using multivariate regression analysis adjusted for relevant confounders. The difference in change in HRQoL between participants with total protein intakes of < 1.0 g/kg/day, intakes of between 1.0–1.2 g/kg/day and intakes of > 1.2 g/kg/day were assessed using one-way ANCOVA.

**Results:**

Total protein intake at baseline was not associated with 12-year changes in physical component summary (PCS) or mental component summary (MCS) scores of HRQoL. Higher animal, red meat and processed animal protein intakes were associated with deteriorations in PCS scores after adjusting for relevant confounders (β = − 0.04; 95% CI: − 0.07, −0.01 ; *p* = 0.009; β = − 0.05; 95% CI: − 0.08, − 0.01; *p* = 0.018; β = − 0.17; 95% CI: − 0.31, − 0.02; *p* = 0.027 respectively). Higher red meat protein intake was associated with deteriorations in MCS scores after adjusting for relevant confounders (β = − 0.04; 95% CI: − 0.08, − 0.01; *p* = 0.011). There was no difference in 12-year changes in PCS or MCS between participants consuming total protein of < 1.0 g/kg/day, 1.0–1.2 g/kg/day and intakes of > 1.2 g/kg/day.

**Conclusion:**

There was no relationship between total dietary protein intake and HRQoL, but higher protein intakes from animal, red meat and processed animal sources were associated with a deterioration in HRQoL scores over 12 years. Due to the number of associations examined and high drop out of older less healthy participants, further research is required to confirm the associations detected in healthy and less healthy participants, with a view to making protein intake recommendations for older adults.

**Supplementary Information:**

The online version contains supplementary material available at 10.1186/s12877-022-02894-y.

## Introduction

Greater life expectancy is driving an increased proportion of adults aged 60 years and older, both globally and in Australia [1–3]. The maintenance of good health, to complement greater life expectancy, represents a challenge due to the higher risk of chronic disease associated with increased age [4]. Maintenance of health-related quality of life (HRQoL), which is a multi-dimensional concept that considers wellbeing and function in physical, mental and social domains, is a key indicator of healthy ageing [5]. A number of modifiable lifestyle behaviours [6–9], including nutrition [10, 11], have the potential to influence HRQoL in older adults. A recent systematic review of observational and intervention studies found that in older adults, healthy dietary patterns were associated with better HRQoL in one or both of physical and mental domains [12].

Adequate dietary protein intake (at least 1.0–1.2 g/kg/day) is recommended for adults aged over 65 years to optimise muscle health and function, and to support recovery from illness [13]. In turn, muscle mass and physical performance have been positively associated with higher HRQoL [14, 15]. Conversely, research also suggests that higher protein intakes, in particular, animal protein, may increase the risk of cardiometabolic risk factors such as Type 2 diabetes [16, 17], which may in turn detrimentally impact HRQL [18]. Hence interest exists in the relationship between dietary protein and HRQoL in older adults. In a cross-sectional study in 83 non-institutionalized older adults (mean age 86 ± 5 years), higher total protein intakes (mean 64 g/day) were associated with lower levels of pain/discomfort assessed via the EuroQoL EQ-5D questionnaire [19]. However, an important limitation is that there was no adjustment for relevant confounders, including age, sex and physical activity. In contrast, three other cross-sectional studies in generally healthy, free-living adults aged 60 years and older found no association between total dietary protein intake and HRQoL [20–22], which may have been due to the high (adequate) protein intakes [mean 72 to 79 g/day (1.1 to 1.4 g/kg/day)] of participants in these studies [20–22] To date, no longitudinal studies have investigated the relationship between total habitual dietary protein intake and any measure of HRQoL in older adults.

Several intervention studies have assessed the effect of different protein sources (alone or with exercise) on HRQoL and measures of muscle strength and function [23–31]. In a 4-month randomised controlled trial (RCT) in women aged 60–90 years, consumption of lean red meat most days of the week combined with progressive resistance training (PRT) improved the physical component summary (PCS) score from the Short-form health survey (SF-36) compared to a carbohydrate rich diet combined with PRT, but there were no differences in the mental component summary (MCS) score [23]. In addition, this study found that changes in lower leg muscle strength were positively associated with changes in HRQoL. A number of other intervention studies have tested the effect of dairy based liquid supplements or low-fat milk alone or with exercise, in adults aged 50 years and older [24–31], with most reporting no beneficial effects on HRQoL. For instance, a 6-month RCT in adults aged over 70 years found that a dairy whey protein supplement combined with strength and balance exercises had no effect on HRQoL when compared to exercise alone [25], despite the whey protein enriched supplement augmenting the effects of exercise on gains in muscle strength and body composition [25, 32]. A 6-month milk protein supplementation trial in frail adults aged 65 years and older that led to improvements in gait speed and chair rise time also failed to improve HRQoL PCS scores [29]. It is possible that the lack of any improvements in HRQoL in many of these studies relates to the lack of or modest improvements in muscle strength or function with the provision of additional protein, or the HRQoL tools used not being sensitive enough to detect changes over the intervention period. Collectively, evidence examining the effects of increased dietary protein, irrespective of source, on HRQoL remains mixed.

Prospective epidemiological cohort studies provide an opportunity to examine the relationship of habitual total protein intake and different protein sources with changes in HRQoL over time. Therefore, the aim of this study was to examine the associations of total dietary protein intake and protein source with changes in HRQoL over a 12-year period in Australian adults aged 60 years and older at baseline.

## Methods

### Participants and setting

**Fig. 1:**
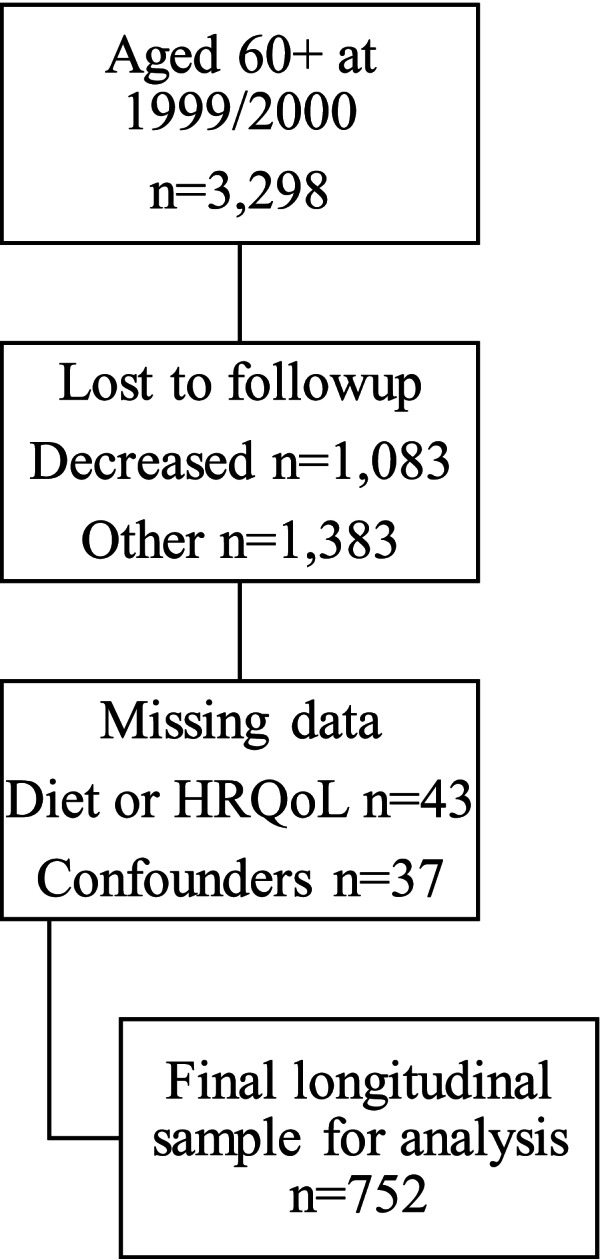
Flowchart of sample for analysis

Participants were from the Australian Diabetes, Obesity and Lifestyle (AusDiab) study [33]. Details of recruitment methods and baseline response rates have been described previously [33]. In brief, AusDiab was a population-based 12-year longitudinal study involving 11,247 community-dwelling Australian adults aged 25 years and older at baseline (1999/2000) [33]. Recruitment was based on 42 randomly selected clusters using census collector districts and stratified by Australian states and territories. The current study used a subset of AusDiab participants aged 60 years and older who had data on baseline dietary intake, all confounders and HRQoL scores for PCS and MCS at both baseline and the 12-year follow-up. Participants with incomplete data were excluded. Of the 3298 AusDiab participants aged 60 years and older at baseline, 2215 (67%) were alive at the 12-year follow-up. Of these 2215 participants, 752 (33%) provided the required data at baseline and the 12-year follow-up and therefore were included in this study. A flowchart of the final sample analysed is shown in Fig. 1.

The AusDiab study was approved by the International Diabetes Institute ethics committee and Alfred Health ethics committees. All AusDiab participants provided written informed consent. The current analysis was approved by Deakin University Human Research Ethics Committee (Project number 2019–222).

### Measurements

Measurements including HRQoL and dietary intake were taken in 1999/2000 and in 2011/2012 (12-year follow-up).

#### Habitual dietary intake

Habitual dietary intake was assessed via self-administration of a 74-item food frequency questionnaire (FFQ). The FFQ was developed in Australia to assess the habitual dietary intakes of an ethnically diverse cohort aged 40–69-years [34]. Participants were asked to indicate how frequently over the preceding 12 months they had consumed a range of foods and beverages. Nutrient intakes from the 12-month FFQ have been validated against weighed food records (of between 3 and 7 days) with results reported previously [34–36]. Habitual daily intakes were calculated based on frequencies and the macronutrient/energy content of each food using NUTTAB95 food composition data [37]. Habitual daily intake for each protein source was calculated by combining protein intake in grams/day of foods with the same protein source as follows: red meat (beef, veal, lamb, pork), processed animal (bacon, luncheon meats, sausages, frankfurters), other animal (meat pies, pasties, quiche, other savoury pastries, pizza, hamburger), full-fat dairy (full-cream milk, yoghurt, all cheeses except for low-fat cheese), low-fat dairy (reduced-fat milk, skim milk, low-fat cheese), soy (soya milk, tofu), vegetable (nuts, peanuts, nut paste, peas, green beans, bean sprouts, baked beans, chickpeas, lentils). Plant protein was calculated by combining soy and vegetable. Dairy protein was calculated by combining full-fat dairy and low-fat dairy. Animal protein was calculated by combining red meat, dairy, fish (steamed, grilled, baked, fried, tinned), chicken, butter, eggs, flavoured milk and ice-cream. Total protein intake was calculated by combining protein from all foods.

#### HRQoL

Health-related quality of life (HRQoL) was collected via the self-administered SF-36 Version 1 questionnaire used with permission from the Medical Outcomes Trust (Boston, MA, USA) [38]. Based on the answers from this 36-question survey, the two summary scores of PCS and MCS were calculated using published guidelines [39, 40]. Summary scores were then normalised to have a mean of 50 and standard deviation of 10 across the Australian general population [39, 40]. Higher scores indicate better HRQoL. The changes in PCS and MCS between baseline and the 12-year follow-up were calculated as the differences in the normalised summary score from baseline to 12 years. Positive results indicate improved HRQoL. The SF-36 has demonstrated good construct validity, test-retest reliability and internal consistency, and has been validated for use in older adults [41–43].

#### Variables considered as confounders

Confounders including age, sex, education, smoking status, urban/rural classification and household type were collected via interview administered questionnaire [33]. Education was based on each participants highest qualification and categorised as: 1) “secondary”, which comprised secondary school qualification; 2) “diploma”, which comprised nursing qualification, teaching qualification, trade certificate, technicians’ certificate, certificate other than above, associate diploma, undergraduate diploma or 3) “degree”, which comprised bachelor’s degree, postgraduate diploma or masters/doctorate. Self-report smoking status was categorised as “current daily smokers”, “ex-smokers” or “never smoked daily”, with “ex-smokers” defined as participants smoking less than daily for at least the last 3 months, but previously smoked daily. Urban/rural classification was based on the census district where the participant resided, with “urban” defined as capital city and “rural” defined as non-capital city. Household type was classified as either “person living alone”, “married or de facto couple only”, “married or de facto couple living with children”, “one person living with children”, “shared household” or “all other households”. Prior history of cardiovascular disease (CVD; angina, coronary heart disease, or stroke) was obtained by self-reported medical history [44]. The presence of diabetes was assigned based on self-report of taking hypoglycaemic medication or on having fasting plasma glucose ≥7.0 mmol/L or a 2-h plasma glucose ≥11.1 mmol/L [44].

Diet quality was assessed using the Dietary Guideline Index (DGI) [45] based on food intakes collected from the 74-item FFQ. The DGI is a food-based dietary index which assesses dietary intake against the 2013 Australian Dietary Guidelines [46]. Indicators of each dietary guideline were identified, with age and sex specific cut-offs developed. The DGI included 11 items: vegetables, fruits, grains/cereals, meats/meat alternatives, dairy/dairy alternatives, discretionary foods, saturated fats, unsaturated fats, diet variety, sugar and alcohol. Two items usually included in the DGI (fluid intake and limiting intake of salty food) were not included, as the FFQ did not collect this data. Adherence was scored from 0 (not meeting recommendation) to 10 (fully meeting recommendation). Total scores ranged from 0 to 110, with higher scores indicating greater diet quality.

Height was measured without shoes to the nearest 0.5 cm using a stadiometer [47]. Weight was measured to the nearest 0.1 kg without shoes, excess clothing or items in pockets, using a mechanical beam balance [47]. Body mass index (BMI) was calculated as weight in kilograms divided by height in meters squared.

Physical activity level was assessed using the validated Active Australia survey [48, 49]. Time spent performing leisure time physical activity (duration and frequency) was reported over the preceding week. Total physical activity was calculated as the sum of the time spent walking for a minimum of 10 min, the time spent performing moderate-intensity activities plus double the time spent performing vigorous physical activities. Because vigorous-intensity activity is commonly considered to contribute additional health benefits, double the time spent in vigorous physical activity is used when creating insufficient and sufficient categories of physical activity. To avoid over-reporting, where the reported time for an activity exceeded 840 min/day, the time spent on the activity was recorded as 840 min/day [49]. Total physical activity was classified as either “none”, “insufficient: 1-149 minutes/week” or “sufficient: ≥150 min/week”.

To facilitate adjustment to the model for energy intake misreporting, the energy misreporting ratio (EI:EE) was calculated as total daily energy intake reported in kilojoules divided by the predicted daily total energy expenditure based on previously published equations and assuming a “low” physical activity level [50].

Data for all potential confounders were collected at baseline, except household type which was collected at the 12-year follow-up.

### Statistical analysis

Descriptive statistics were presented as mean ± standard deviation (SD) for continuous data or number and percentage for categorical data. Differences between included and excluded participants were assessed using independent sample *t*–tests for continuous variables and chi-squared tests for categorical variables. Changes in HRQoL from baseline to the 12-year follow-up were assessed using paired *t*-tests. The interaction of the relationship between protein intakes and HRQoL by sex was assessed using linear regression.

The associations between baseline total dietary protein in g/kg/day and baseline protein sources in g/day and changes in PCS and MCS over 12 years were assessed using multivariate regression analysis adjusted for relevant confounders. Grams/kg/day were used to assess total protein as recommended total protein intakes are generally provided in g/kg/day [13]. The difference in change in PCS and MCS over 12 years between participants with total protein intakes of < 1.0 g/kg/day, intakes of between 1.0–1.2 g/kg/day and intakes of > 1.2 g/kg/day were assessed using one-way ANCOVA. Protein intake cut-points were chosen based on the recommendation from the PROT-AGE study group that adults aged over 65-years consume dietary protein of at least 1.0–1.2 g/kg/day [13]. The selection of confounders was based on evidence in the literature of a confounder’s association with both protein intake and HRQoL. Directed acyclic graphs [51] were used to assist with the identification of key confounders based on assumed directions of associations between covariates, the exposure and the outcome (Supplemental Fig. 1). The confounders included in model 1 were age, sex, education, physical activity, household type and urban/rural classification. Model 2 included all confounders included in model 1 plus BMI (the direction of the relationship between protein intake and BMI is unclear i.e. protein intake may influence BMI or BMI may influence protein intake). Based on the literature, the presence of diabetes [52] and CVD [53] were considered to be on the causal pathway between intakes of dietary protein (total protein and different sources of protein) and HRQoL, as was diet quality (as protein intake and protein source are components of diet quality [45]) and therefore not included as confounding factors in the main model [54]. However, sensitivity analysis was performed including diet quality and the presence of diabetes and CVD. To adjust for possible over and under reporting of energy intake, the model also included EI:EE. The possibility of non-linear relationships between protein intakes and 12-year changes in HRQoL was assessed using squared protein intakes. No evidence of non-linearity was found. Residuals from regression models were assessed for normality and heteroscedasticity using P-P plots and plots of residuals against fitted values, respectively. To determine the robustness of our findings, the following sensitivity analyses were performed. In the first sensitivity analysis, extreme energy intake reporters (defined as EI:EE outside mean EI:EE ± 1 SD) were excluded from the model [55]. In the second sensitivity analysis, baseline HRQoL was included in the model as a covariate. In the third sensitivity analysis, participants baseline CVD and diabetes status, together with diet quality, were included in the model as confounders. Statistical analysis was performed using SPSS Software (version 25, 2017, IBM Corp). Significance was defined as *p* < 0.05. A sample size of *n* = 752 provides 80% power in linear regression analyses to detect squared partial correlations as small as 0.01.

## Results

Baseline characteristics and nutrient intakes of the 752 participants are shown in Table 1. The mean (±SD) age of participants was 66.1 ± 5.0 years and 51.3% of the participants were female. Mean (±SD) total daily protein intake/kg was 1.19 ± 0.57 g with 37.5% of participants consuming < 1.0 g/kg/day, 22.6% consuming 1.0–1.2 g/kg/day and 39.9% consuming > 1.2 g/kg/day (recommendation from the PROT-AGE study group for adults aged over 65 years ≥1.0–1.2 g/kg/day) [13].

**Table 1:** Baseline characteristics of participants

	Total
	***(n*** **= 752)**
Age (years), mean (SD)	66.1 ± 5.0
Sex (female) n, (%)	386 (51.3%)
BMI (kg/m^2^), mean (SD)	27.0 ± 4.1
- Underweight (< 18.5 kg/m^2^) n, (%)	3 (0.4%)
- Healthy weight (18.5–24.9 kg/m^2^) n, (%)	242 (32.2%)
- Overweight (25.0–29.9 kg/m^2^) n, (%)	353 (46.9%)
- Obese (≥ 30.0 kg/m^2^) n, (%)	154 (20.5%)
Education	
- Secondary n, (%)	326 (43.4%)
- Diploma n, (%)	334 (44.4%)
- Degree n, (%)	92 (12.2%)
Smoking status^a^	
- Current daily smokers n, (%)	40 (5.4%)
- Ex-smokers n, (%)	257 (34.5%)
- Never smoked daily n, (%)	447 (60.1%)
Urban/Rural classification	
- Urban n, (%)	474 (63.0%)
- Rural n, (%)	278 (37.0%)
Physical activity	
- None n, (%)	118 (15.7%)
- Insufficient (1–149 min/week) n, (%)	197 (26.2%)
- Sufficient (≥150 min/week) n, (%)	437 (58.1%)
Prevalence of Cardiovascular disease^a^, n (%)	94 (12.7%)
Prevalence of Diabetes^a^, n (%)	89 (11.9%)
Household type^b^	
- Person living alone, n (%)	227 (30.2%)
- Married or de facto couple only, n (%)	469 (62.3%)
- Married or de facto couple living with children, n (%)	22 (2.9%)
- One person living with children, n (%)	14 (1.9%)
- Shared household, n (%)	18 (2.4%)
- All other households, n (%)	2 (0.3%)
Energy misreporting ratio, mean (SD)	0.88 ± 0.01
Energy (kJ/day), mean (SD)	7650 ± 2906
Total protein (g/kg/day), mean (SD)	1.19 ± 0.57
Total protein (g/day), mean (SD)	88.2 ± 42.2
Animal protein (g/day), mean (SD)	51.3 ± 33.3
Red meat protein (g/day), mean (SD)	18.8 ± 23.8
Processed animal protein (g/day), mean (SD)	3.8 ± 5.9
Other animal protein (g/day), mean (SD)	2.9 ± 2.7
Dairy protein (g/day), mean (SD)	16.6 ± 8.6
Full-fat dairy protein (g/day), mean (SD)	7.4 ± 6.1
Low-fat dairy protein (g/day), mean (SD)	9.3 ± 9.0
Plant protein (g/day), mean (SD)	3.0 ± 3.3
Soy protein (g/day), mean (SD)	0.8 ± 2.6
Vegetable protein (g/day), mean (SD)	2.2 ± 1.9
Dietary Guideline Index, mean (SD)	69.3 ± 13.0
Total fat (g/day), mean (SD)	71.9 ± 34.0
Total carbohydrate (g/day), mean (SD)	208.8 ± 75.2

*SD* Standard deviation, *BMI* Body mass index, ^a^ Data not complete, percentage based on valid responses (variable included for information only, not treated as a covariate), ^b^ Data collected at 12-year follow-up. Note: The sum of animal and plant protein sources differs from total protein as total protein includes protein from all sources (e.g. includes cereals and fruit).

Compared to the 752 participants included in this study, the 2546 excluded were older, had a higher BMI, were more likely to be from a rural location, had a higher prevalence of CVD and diabetes, had lower PCS and MCS scores, had lower levels of education, had a higher proportion of current smokers and had lower levels of physical activity (Supplemental Table 1).

HRQoL scores deteriorated significantly after 12 years for both PCS (baseline 47.8 ± 8.6 versus 12-year 42.1 ± 10.6; *p* < 0.001) and MCS scores (baseline 52.2 ± 8.5 versus 12-year 51.4 ± 9.1; *p* = 0.015).

### Protein intakes and changes in HRQoL over 12 years

Analysis of the interaction between sex, protein intake and HRQoL found limited interactions (significant in only two of the 20 relationships assessed) (data not shown). Therefore, data for males and females were pooled.

Table 2 shows the regression models examining the association between baseline protein intakes by source in grams/day and 12-year changes in HRQoL, with the β-coefficients representing the expected change in HRQoL per one additional gram of protein. In the fully adjusted model, higher intakes of animal protein, red meat protein and processed animal protein were associated with detrimental changes in PCS scores. That is, for every additional 10 g of protein there was a 0.4 (animal protein), 0.5 (red meat protein) and 1.7 (processed meat protein) deterioration in the 12-year change in PCS scores. Higher intakes of red meat protein were also associated with detrimental changes in MCS in the fully adjusted model. For every additional 10 g of red meat protein there was a 0.4 deterioration in MCS scores over 12-years.

**Table 2:** Associations between baseline protein intake and 12-year changes in health-related quality of life

			Total (***n*** = 752)			
	Unadjusted		Model 1^*^		Model 2^**^	
	β (CI)	***P***-value	β (CI)	***P***-value	β (CI)	***P***-value
**Change in PCS score**						
Animal protein	− 0.01 (− 0.03, 0.02)	0.597	***−0.05 (− 0.08, − 0.02)***	***0.003***	***− 0.04 (− 0.07, − 0.01)***	***0.009***
Red meat protein	−0.01 (− 0.04, 0.02)	0.509	***− 0.05 (− 0.08, − 0.01)***	***0.010***	***− 0.05 (− 0.08, − 0.01)***	***0.018***
Processed animal protein	−0.05 (− 0.17, 0.07)	0.383	***−0.17 (− 0.31, − 0.03)***	***0.021***	***− 0.17 (− 0.31, − 0.02)***	***0.027***
Other animal protein	0.06 (− 0.20, 0.32)	0.638	−0.09 (− 0.36, 0.18)	0.508	− 0.08 (− 0.35, 0.19)	0.574
Dairy protein	− 0.06 (− 0.14, 0.02)	0.166	−0.06 (− 0.15, 0.02)	0.135	−0.06 (− 0.15, 0.02)	0.146
Full-fat dairy protein	− 0.11 (− 0.22, 0.01)	0.068	− 0.11 (− 0.22, 0.01)	0.065	−0.12 (− 0.23, 0.00)	0.051
Low-fat dairy protein	0.00 (− 0.08, 0.07)	0.930	− 0.01 (− 0.08, 0.07)	0.892	0.00 (− 0.08, 0.08)	0.975
Plant protein	***0.24 (0.03, 0.45)***	***0.023***	0.18 (− 0.03, 0.39)	0.101	0.17 (− 0.04, 0.38)	0.109
Soy protein	0.14 (−0.13, 0.42)	0.308	0.12 (−0.15, 0.39)	0.396	0.11 (−0.16, 0.38)	0.439
Vegetable protein	***0.50 (0.13, 0.88)***	***0.008***	0.37 (−0.03, 0.76)	0.068	0.37 (−0.02, 0.77)	0.062
**Change in MCS score**						
Animal protein	***−0.02 (− 0.04, 0.00)***	***0.018***	***− 0.04 (− 0.07, − 0.01)***	0.004	− 0.03 (− 0.06, 0.00)	0.060
Red meat protein	***− 0.04 (− 0.07, − 0.10)***	***0.003***	***−0.06 (− 0.09, − 0.02)***	***0.001***	***− 0.04 (− 0.08, − 0.01)***	***0.011***
Processed animal protein	−0.10 (− 0.20, 0.01)	0.085	− 0.11 (− 0.24, 0.03)	0.129	− 0.08 (− 0.22, 0.05)	0.236
Other animal protein	0.16 (− 0.07, 0.40)	0.167	0.20 (− 0.05, 0.44)	0.119	0.24 (− 0.01, 0.48)	0.061
Dairy protein	−0.01 (− 0.08, 0.07)	0.866	0.00 (− 0.08, 0.8)	0.947	0.00 (− 0.08, 0.08)	0.958
Full-fat dairy protein	− 0.01 (− 0.12, 0.09)	0.799	0.00 (− 0.10, 0.11)	0.961	−0.01 (− 0.12, 0.09)	0.811
Low-fat dairy protein	0.00 (− 0.07, 0.07)	0.991	0.00 (− 0.08, 0.07)	0.925	0.01 (− 0.06, 0.08)	0.835
Plant protein	−0.03 (− 0.22, 0.16)	0.783	− 0.03 (− 0.22, 0.17)	0.801	− 0.04 (− 0.23, 0.16)	0.714
Soy protein	0.09 (− 0.16, 0.33)	0.494	0.06 (− 0.19, 0.31)	0.614	0.04 (− 0.21, 0.28)	0.775
Vegetable protein	−0.24 (− 0.58, 0.09)	0.155	−0.22 (0.59, 0.14)	0.227	−0.20 (− 0.56, 0.16)	0.270

β represents the expected change in HRQoL with 1 additional gram of protein. CI - 95% confidence interval; *PCS* Physical component summary, *MCS* Mental component summary, ^*^ Model 1 adjusted for age, sex, education, physical activity, urban/rural classification, household type and energy misreporting ratio; Model 2 adjusted for all confounders included in Model 1 plus BMI

Regression examining the association between baseline total protein intakes in grams/kg/day and 12-year changes in HRQoL found no relationship in either the unadjusted or the fully adjusted model for either PCS or MCS scores. In the unadjusted model the results for PCS were β = 0.47; 95% CI: − 0.75, 1.70; *p* = 0.451 and MCS β = − 0.41; 95% CI: − 1.52, 0.70; *p* = 0.465. In the fully adjusted model, results for PCS were β = − 1.77; 95% CI: − 4.49, 0.95; *p* = 0.202 and MCS β = 0.41; 95% CI: − 3.55, 1.44; *p* = 0.406.

### Difference in HRQoL between categories of total protein intake

One-way ANCOVA found no change in the 12-year PCS (*p* = 0.269) and MCS (*p* = 0.510) scores across the three categories of total protein intake (< 1.0 g/kg/day, 1.0–1.2 g/kg/day, > 1.2 g/kg/day).

### Sensitivity analysis

Sensitivity analysis supported results from the main analysis. In all sensitivity analyses, detrimental associations between animal and red meat proteins and PCS were confirmed. The detrimental association between red meat protein and MCS was confirmed in two of the three sensitivity analyses (Supplemental Table 2). When extreme energy reporters were excluded, a detrimental association between total protein and PCS was detected (β = − 4.61; 95% CI: − 8.92, − 2.90; *p* = 0.037). There were no other changes in results between total protein and HRQoL (results not shown).

## Discussion

The main finding from this 12-year prospective study in Australian adults aged 60 years and older was that higher intakes of red meat protein were associated with detrimental changes in both PCS and MCS, while animal protein and processed animal protein were associated with detrimental changes in PCS. Total dietary protein, dairy protein and plant protein were not associated with changes in HRQoL. Moreover, there was no difference in changes in HRQoL between participants who exceeded the total recommended protein intake compared with those who met the recommendation and those consuming below the recommended intake.

In this 12-year longitudinal study we found that total dietary protein was not associated with changes in HRQoL. To our knowledge, our study is the first to investigate the long-term association between habitual dietary protein intake with changes in HRQoL. However, our study’s results are consistent with several previous cross-sectional studies [20–22] and short-term RCTs (duration ≤6-months) [24–31] in healthy adults aged 50 years and older which have similarly reported no association between total protein intake and HRQoL. For instance, Ten Haaf et al. [20] reported no association between total protein intake and any measure of HRQoL in 140 adults aged 81 ± 6 years.

The finding in our study that red meat protein intake was associated with detrimental changes in HRQoL contrasts with the evidence from a 4-month intervention in healthy community-dwelling women aged 60–90 years which reported a beneficial effect of increased lean red meat intake with PRT on PCS, but not MCS, when compared with a control group consuming carbohydrate combined with PRT [23]. However, it is difficult to compare these findings with the current study due to the different study designs (intervention vs prospective epidemiologic study), participant numbers (100 vs 752), follow-up periods (4 months vs 12 years), the inclusion of PRT and the provision of high quality lean red meat in the aforementioned intervention. There is, however, some evidence that red meat consumption may play a role in the risk of depression with both intakes above 57 g/day and below 28 g/day associated with increased rates of depression independent of diet quality in a cohort of female Australian adults [56]. This is of relevance to our study as there is evidence to support a strong association between depression and HRQoL [57].

A novel finding from our study was that meat-based proteins (red meat protein and processed animal protein) were associated with detrimental changes in PCS. Recent evidence suggests several detrimental health outcomes associated with higher meat-based protein intakes. For instance, higher meat intake, and a higher animal protein to vegetable protein intake ratio were both associated with an increased risk of mortality in a 22-year longitudinal study of 2641 Finnish males aged 42–60 years [58]. Consumption of processed meat has also been associated with numerous chronic health conditions, including colorectal cancer, coronary heart disease and diabetes [59–61]. Thus, the presence of chronic conditions could explain, at least in part, the relationships observed between meat-based proteins and the deterioration in PCS in the current study.

Another possible explanation for the associations detected between meat-based proteins and detrimental changes in PCS is that the saturated fat associated with meat-based proteins has caused the detrimental effect on PCS. Diets high in saturated fat produce a less diverse and more inflammatory gut microbiome [62], and increased systemic inflammation which has been linked to many age-related diseases such as rheumatoid arthritis, sarcopenia (muscle loss) and osteoporosis [63]. Thus, it could be hypothesized that higher consumption of saturated fat by consuming higher meat-based proteins may have increased rates of these age-related diseases. The association between higher saturated fat intake and lower PCS has been observed in previous observational studies [64, 65]. We considered treating saturated fat as a covariate, however due to the high correlation between total protein and saturated fat (ρ = 0.83) there were concerns that this would impact the validity of the model’s results [66]. The inability to control for saturated fat is a limitation of the findings. Nevertheless, it is worth noting that despite the significant adverse relationships between increased meat-based protein intake and changes in HRQoL, the associations were modest. For instance, every additional 10 g of red meat protein was associated with a detrimental 12-year change in PCS scores of 0.5, while changes in HRQoL scores of between three and five are generally considered clinically meaningful [67, 68].

Recent clinical and consensus guidelines have recommended that the total protein intake for adults aged over 65 years be at least 1.0–1.2 g/kg/day in order to optimise muscle health and function, and to support recovery from illness [13]. In our study, we found that changes in HRQoL in participants with total protein intakes below recommendations did not differ from those with protein intakes at or above recommendations. These findings suggest that there is no specific protein intake cut-point (when expressed as g/kg/day) that is associated with HRQoL.

A number of limitations must be considered when interpreting these findings. Firstly, a limitation of this study, as well as previous observational studies on this topic [19–22], is the modest sample size of 752. Secondly, although this study included a range of confounders, it is possible residual confounding remained because of unmeasured confounders. Thirdly, associations were only assessed using baseline protein intakes and confounders. Protein intake at baseline was used to reduce the potential of reverse causation (for example a reduction in levels of HRQoL over 12 years leading to a change in dietary intake at follow-up) and because food consumption patterns in older adults tend to be stable [69]. It is, however, possible that changes in protein intakes and confounders which influence HRQoL have occurred over the study’s 12-year duration. Fourthly, only community-dwelling adults were eligible to participate in the AusDiab study, and thus the results cannot be generalised to other populations. Fifthly, the study was exploratory in its analysis of a range of protein sources and therefore correction for multiple comparisons was not employed. Caution should be used when interpreting the results of this study due to the number of associations assessed with no adjustments made for multiple comparisons, which may increase the likelihood of a type I error. The results of this study provide a hypothesis of associations which need to be corroborated by future research. Finally, it is possible that our study’s results are impacted by selection bias due to high dropout rates and exclusion of participants with incomplete data. The age of participants (60 years and older at baseline), together with this study’s long-term duration, have contributed to only 23% of eligible participants providing dietary data at the 12-year follow-up and therefore being included in the main analysis. Of note, 33% of eligible participants were deceased at the 12-year follow-up. Analysis revealed that included participants appeared to be “healthier” (lower prevalence of CVD and diabetes, lower rates of smoking and physical inactivity, lower BMI and higher baseline HRQoL) than excluded participants. To determine the potential impact of excluding “less healthy” participants, analysis was performed to determine the relationship between baseline HRQoL and change in 12-year HRQoL. Pearson’s correlation revealed a moderate negative correlation (PCS ρ = − 0.33, MCS ρ = − 0.45), indicating that higher baseline HRQoL was associated with larger declines in HRQoL. This suggests our results may only be generalizable to healthier participants. However, it should also be noted that sensitivity analysis revealed only a marginal decrease in the association between protein intake and HRQoL when baseline HRQoL was included in the model. Despite the low number of participants available for our analysis, there are a number of strengths to the original AusDiab study which is why it was used for our secondary analysis. These include the AusDiab’s prospective design and 12-year follow-up period which has allowed long-term associations to be investigated. In addition, the AusDiab study used validated tools to measure dietary data and HRQoL.

In conclusion, this 12-year longitudinal study in Australian adults aged 60 years and older found that total dietary protein was not associated with changes in HRQoL, while higher animal, red meat and processed animal protein intakes were associated with detrimental changes in either PCS and/or MCS scores. We found that meeting recommended daily total protein intakes (when expressed as grams per kg) did not influence 12-year HRQoL. Our results suggest that clinical advice, to potentially minimise long-term detrimental effects to HRQoL, include recommendations on avoiding animal protein, red meat protein and processed animal protein when choosing proteins to consume. Dietary guidelines for older adults should consider protein source when advising older adults on protein consumption.

## Supplementary Information

**Additional file 1: Supplemental Fig. 1** Diagrammatic interpretation of the relationship between potential confounders, exposure and outcome. **Supplemental Table 1** Baseline characteristics of participants included versus excluded. **Supplemental Table 2** Sensitivity analysis: Associations between baseline protein intake by source in grams per day and 12-year changes in health-related quality of life.

## Data Availability

The data that support the findings of this study are available from the Australian Diabetes, Obesity and Lifestyle study, contact Prof. Jonathan Shaw (Baker Heart and Diabetes Institute), but restrictions apply to the availability of these data, which were used under license for the current study, and so are not publicly available.

## Abbreviations

AusDiab: Australian Diabetes, Obesity and Lifestyle study
BMI: Body mass index
CI: Confidence interval
CVD: Cardiovascular disease
DGI: Dietary Guideline Index
EI:EE: energy misreporting ratio
FFQ: Food frequency questionnaire
HRQoL: Health-related quality of life
MCS: Mental component summary
PCS: Physical component summary
PRT: Progressive resistance training
RCT: Randomised controlled trial
SF-36: Short-form health survey
SD: Standard deviation
