# Supplementary material for: Association between dietary protein intake and changes in health-related quality of life in older adults: findings from the AusDiab 12-year prospective study
Source: BMC Geriatr. 2022 Mar 16;22:211. doi: 10.1186/s12877-022-02894-y (PMC8925096; doi:10.1186/s12877-022-02894-y)
Supplement: Supplementary file 1 — Additional file 1: Supplemental Fig. 1 Diagrammatic interpretation of the relationship between potential confounders, exposure and outcome. Supplemental Table 1 Baseline characteristics of participants included versus excluded. Supplemental Table 2 Sensitivity analysis: Associations between baseline protein intake by source in grams per day and 12-year changes in health-related quality of life. [file 12877_2022_2894_MOESM1_ESM.docx]

**Supplemental Figure 1** Diagrammatic interpretation of the relationship between potential confounders, exposure and outcome


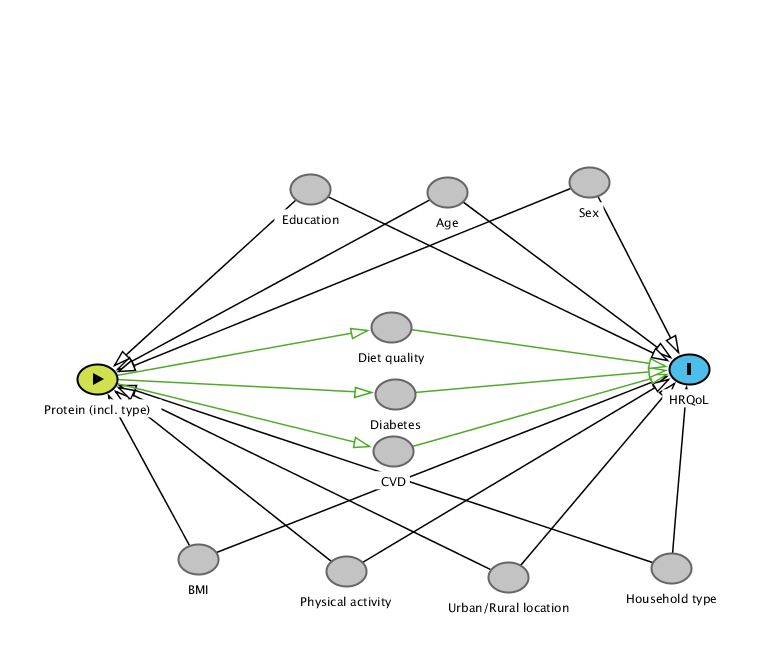


**Supplemental Table 1** Baseline characteristics of participants included versus excluded

|  | **Included** | **Excluded**^c^ |  |
| --- | --- | --- | --- |
|  | ***(n*= 752)** | ***(n*= 2546)** | ***P-value***^*^ |
| Age (years), mean (SD) | 66.1 ± 5.0 | 70.8 ± 6.9 | <0.001 |
| Sex (female) n (%) | 386 (51.3%) | 1390 (54.6%) | 0.124 |
| BMI (kg/m^2^), mean (SD) | 27.0 ± 4.1 | 27.4 ± 4.7 | 0.015 |
| Education |  |  |  |
| - Secondary n, (%) | 326 (43.4%) | 1431 (57.1%) | <0.001 |
| - Diploma n, (%) | 334 (44.4%) | 923 (36.9%) |  |
| - Degree n, (%) | 92 (12.2%) | 151 (6.0%) |  |
| Smoking status^a^ |  |  |  |
| - Current daily smokers n, (%) | 40 (5.4%) | 231 (9.3%) | 0.001 |
| - Ex-smokers n, (%) | 257 (34.5%) | 882 (35.5%) |  |
| - Never smoked daily n, (%) | 447 (60.1%) | 1369 (55.2%) |  |
| Urban/Rural classification |  |  |  |
| - Urban n, (%) | 474 (63.0%) | 1388 (54.5%) | <0.001 |
| - Rural n, (%) | 278 (37.0%) | 1158 (45.5%) |  |
| Physical activity |  |  |  |
| - None n, (%) | 118 (15.7%) | 526 (20.8%) | <0.001 |
| - Insufficient (1-149 minutes/week) n, (%) | 197 (26.2%) | 786 (31.2%) |  |
| - Sufficient (≥150 minutes/week) n, (%) | 437 (58.1%) | 1209 (48.0%) |  |
| Prevalence of Cardiovascular disease^a^, n (%) | 94 (12.7%) | 616 (24.7%) | <0.001 |
| Prevalence of Diabetes^a^ ,n (%) | 89 (11.9%) | 542 (21.6%) | <0.001 |
| Household type^b^ |  |  |  |
| - Person living alone, n (%) | 227 (30.2%) | 13 (26.0%) | 0.569 |
| - Married or de facto couple only, n (%) | 469 (62.3%) | 32 (64.0%) |  |
| - Married or de facto couple living with children, n (%) | 22 (2.9%) | 2 (4.0%) |  |
| - One person living with children, n (%) | 14 (1.9%) | 0 (0%) |  |
| - Shared household, n (%) | 18 (2.4%) | 3 (6.0%) |  |
| - All other households, n (%) | 2 (0.3%) | 0 (0%) |  |
| Health-related quality of life |  |  |  |
| SF-36 Physical component summary (PCS) score, mean (SD) | 47.8 ± 8.6 | 42.9 ± 10.9 | <0.001 |
| SF-36 Mental component summary (MCS) score, mean (SD) | 52.2 ± 8.5 | 50.2 ± 9.7 | <0.001 |

SD - Standard deviation; BMI - body mass index; ^a^ Data not complete (variable included for information only, not treated as a covariate); ^b^ Data collected at 12-year follow-up; ^c^ figures based on participants who provided valid data; ^*^ *p*-values based on assessments of differences between included and excluded participants using independent sample *t*–tests for continuous variables and chi-squared test for categorical variables

**Supplemental Table 2** Sensitivity analysis: Associations between baseline protein intake by source in grams per day and 12-year changes in health-related quality of life

|  | **Sensitivity 1**^*^ | | **Sensitivity 2**^**^ | | **Sensitivity 3**^***^ |  |
| --- | --- | --- | --- | --- | --- | --- |
|  | **(*n*=602)** |  | **(*n*=752)** |  | **(*n*=752)** |  |
|  | **β (CI)** | **P-value** | **β (CI)** | **P-value** | **β (CI)** | **P-value** |
| **Change in PCS score** |  |  |  |  |  |  |
| Animal protein | ***-0.08 (-0.13, -0.04)*** | ***0.001*** | ***-0.04 (-0.06, -0.01)*** | ***0.014*** | ***-0.05 (-0.08, -0.02)*** | ***0.002*** |
| Red meat protein | ***-0.09 (-0.15, -0.03)*** | ***0.003*** | ***-0.04 (-0.07, 0.00)*** | ***0.027*** | ***-0.05 (-0.09. -0.01)*** | ***0.014*** |
| Processed animal protein | ***-0.35 (-0.61, -0.10)*** | ***0.007*** | -0.11 (-0.24, 0.03) | 0.123 | -0.15 (-0.30, 0.0) | 0.050 |
| Other animal protein | -0.09 (-0.40, 0.23) | 0.593 | -0.11 (-0.36, 0.13) | 0.365 | -0.04 (-0.32, 0.23) | 0.759 |
| Dairy protein | -0.07 (-0.17, 0.03) | 0.151 | -0.07 (-0.15, 0.01) | 0.073 | *-0.10 (-0.19, -0.01)* | *0.039* |
| Full-fat dairy protein | ***-0.14 (-0.28, -0.01)*** | ***0.036*** | -0.10 (-0.20, 0.01) | 0.080 | -0.11 (-0.22, 0.01) | 0.080 |
| Low-fat dairy protein | 0.00 (-0.09, 0.09) | 0.947 | -0.02 (-0.09, 0.05) | 0.634 | -0.03 (-0.11, 0.06) | 0.504 |
| Plant protein | 0.16 (-0.07, 0.39) | 0.180 | 0.10 (-0.10, 0.29) | 0.325 | 0.13 (-0.09, 0.35) | 0.234 |
| Soy protein | 0.05 (-0.23, 0.34) | 0.724 | 0.06 (-0.19, 0.31) | 0.630 | 0.06 (-0.23, 0.34) | 0.698 |
| Vegetable protein | ***0.50 (0.04, 0.97)*** | ***0.034*** | 0.21 (-0.15, 0.57) | 0.254 | 0.33 (-0.07, 0.73) | 0.104 |
| **Change in MCS score** |  |  |  |  |  |  |
| Animal protein | -0.02 (-0.06, 0.02) | 0.364 | -0.02 (-0.04, 0.01) | 0.161 | ***-0.31 (-0.06, 0.00)*** | ***0.036*** |
| Red meat protein | -0.03 (-0.08, 0.03) | 0.330 | ***-0.04 (-0.07, -0.01)*** | ***0.021*** | ***-0.04 (-0.08, -0.01)*** | ***0.011*** |
| Processed animal protein | -0.10 (-0.34, 0.13) | 0.382 | -0.06 (-0.18, 0.06) | 0.343 | -0.08 (-0.22, 0.06) | 0.265 |
| Other animal protein | 0.25 (-0.04, 0.53) | 0.091 | 0.17 (-0.06, 0.39) | 0.143 | ***0.26 (0.01, 0.51)*** | ***0.042*** |
| Dairy protein | -0.04 (-0.13, 0.05) | 0.348 | 0.04 (-0.03, 0.11) | 0.300 | -0.01 (-0.09, 0.07) | 0.844 |
| Full-fat dairy protein | -0.05 (-0.17, 0.08) | 0.455 | -0.02 (-0.11, 0.08) | 0.753 | -0.01 (-0.11, 0.10) | 0.922 |
| Low-fat dairy protein | -0.02 (-0.09, 0.07) | 0.713 | 0.04 (-0.03, 0.10) | 0.243 | 0.00 (-0.08, 0.07) | 0.909 |
| Plant protein | -0.03 (0.24, 018) | 0.179 | -0.04 (-0.22, 0.13) | 0.627 | -0.05 (-0.25, 0.16) | 0.648 |
| Soy protein | 0.03 (-0.23, 0.29) | 0.833 | 0.03 (-0.19, 0.25) | 0.800 | 0.04 (-0.22, 0.30) | 0.759 |
| Vegetable protein | -0.20 (-0.62, 0.22) | 0.351 | -0.21 (-0.53, 0.11) | 0.201 | -0.24 (-0.61, 0.13) | 0.202 |

^*^ Sensitivity 1: Excluding energy intake misreporters (defined as outside the range mean energy expenditure: energy intake ratio ±1 standard deviation) model adjusted for age, sex, BMI, education, physical activity, urban/rural classification, household type and energy misreporting ratio ^**^ Sensitivity 2: Baseline HRQoL added to the model – Model adjusted for age, sex, BMI, education, physical activity, urban/rural classification, household type, energy misreporting ratio and baseline HRQoL (baseline PCS for change in PCS, baseline MCS for change in MCS); Sensitivity 3: Diet quality, prevalence of cardiovascular disease, prevalence of diabetes added to the model – Model adjusted for age, sex, BMI, education, physical activity, urban/rural classification, household type, energy misreporting ratio, diet quality, prevalence of cardiovascular disease, prevalence of diabetes β represents the expected change in HRQoL with 1 additional gram of protein. CI - 95% confidence interval; PCS - Physical component summary; MCS - Mental component summary
